# Respiratory and Photosynthetic Responses of Antarctic Vascular Plants Are Differentially Affected by CO_2_ Enrichment and Nocturnal Warming

**DOI:** 10.3390/plants11111520

**Published:** 2022-06-06

**Authors:** Carolina Sanhueza, Daniela Cortes, Danielle A. Way, Francisca Fuentes, Luisa Bascunan-Godoy, Nestor Fernandez Del-Saz, Patricia L. Sáez, León A. Bravo, Lohengrin A. Cavieres

**Affiliations:** 1 Laboratorio de Fisiología Vegetal, Departamento de Botánica, Facultad de Ciencias Naturales y Oceanográficas, Universidad de Concepción, Barrio Universitario s/n, Concepción, Casilla 160-C, Concepción 4030000, Chile; lubascun@udec.cl (L.B.-G.); lcaviere@udec.cl (L.A.C.); 2 Laboratorio Cultivo de Tejidos Vegetales, Centro de Biotecnología, Departamento de Silvicultura, Facultad de Ciencias Forestales, Universidad de Concepción, Casilla 160-C, Concepción 4030000, Chile; dancortes@udec.cl (D.C.); franfuentese@udec.cl (F.F.); patrisaez@udec.cl (P.L.S.); 3 Department of Biology, University of Western Ontario, 1151 Richmond Street, London, ON N6A 5B7, Canada; dway4@uwo.ca; 4 Nicholas School of the Environment, Duke University, Durham, NC 27708, USA; 5 Division of Plant Sciences, Research School of Biology, The Australian National University, Canberra, ACT 2601, Australia; 6 Instituto de Ecología y Biodiversidad-IEB, Las Palmeras 3425, Ñuñoa, Santiago 7800003, Chile; 7 Laboratorio de Fisiología y Biología Molecular Vegetal, Instituto de Agroindustria, Departamento de Ciencias Agronómicas y Recursos Naturales, Facultad de Ciencias Agropecuarias y Forestales and Center of Plant, Soil Interaction and Natural Resources Biotechnology, Scientific and Technological Bioresource Nucleus, Universidad de La Frontera, Casilla 54-D, Temuco 4780000, Chile; leon.bravo@ufrontera.cl

**Keywords:** atmospheric CO_2_ concentration, nocturnal warming, respiration, photosynthesis, foliar carbon balance, Antarctic plant species

## Abstract

Projected rises in atmospheric CO_2_ concentration and minimum night-time temperatures may have important effects on plant carbon metabolism altering the carbon balance of the only two vascular plant species in the Antarctic Peninsula. We assessed the effect of nocturnal warming (8/5 °C vs. 8/8 °C day/night) and CO_2_ concentrations (400 ppm and 750 ppm) on gas exchange, non-structural carbohydrates, two respiratory-related enzymes, and mitochondrial size and number in two species of vascular plants. In *Colobanthus quitensis*, light-saturated photosynthesis measured at 400 ppm was reduced when plants were grown in the elevated CO_2_ or in the nocturnal warming treatments. Growth in elevated CO_2_ reduced stomatal conductance but nocturnal warming did not. The short-term sensitivity of respiration, relative protein abundance, and mitochondrial traits were not responsive to either treatment in this species. Moreover, some acclimation to nocturnal warming at ambient CO_2_ was observed. Altogether, these responses in *C. quitensis* led to an increase in the respiration-assimilation ratio in plants grown in elevated CO_2_. The response of *Deschampsia antarctica* to the experimental treatments was quite distinct. Photosynthesis was not affected by either treatment; however, respiration acclimated to temperature in the elevated CO_2_ treatment. The observed short-term changes in thermal sensitivity indicate type I acclimation of respiration. Growth in elevated CO_2_ and nocturnal warming resulted in a reduction in mitochondrial numbers and an increase in mitochondrial size in *D. antarctica*. Overall, our results suggest that with climate change *D. antarctica* could be more successful than *C. quitensis*, due to its ability to make metabolic adjustments to maintain its carbon balance.

## 1. Introduction

Since the Industrial Revolution, atmospheric CO_2_ concentrations have steadily increased, and by the end of the century, CO_2_ concentrations will be >550 ppm as a consequence of fossil fuel burning and land-use changes [1,2]. Consequently, climate warming will likely increase global mean surface temperatures by 1.5 °C between 2030 and 2052 [3], while for high latitudes, such as the Antarctic, two-thirds of climate models project warming of 1.8 °C to 3.3 °C by the year 2100 [4]. Additionally, temperatures are increasing more rapidly during the night than during the day [5], and this asymmetric warming is projected to be stronger at high latitudes where cold nights could warm by up to 4.5 °C [3].

The physiological effects of nocturnal warming and increased atmospheric CO_2_ concentrations on the carbon metabolism, and consequently, performance of plants inhabiting polar ecosystems, are still poorly understood. Plant carbon metabolism is governed by photosynthesis and respiration, which are interdependent processes, where most of the non-structural carbohydrates produced by photosynthesis, including soluble sugars and starch, are used as substrates by respiration [6,7]. While it is well known that net photosynthesis (A_net_) and respiration tend to be correlated [8,9,10], each process is differentially affected by environmental factors. Thus, the impact of warming and/or elevated CO_2_ on plant carbon metabolism will depend on their differential effects on photosynthesis and respiration [11].

Increases in temperature often increase A_net_ by stimulating biochemical reaction rates, including photosynthetic electron transport and Rubisco carboxylation [12,13,14]. However, photosynthetic acclimation to environmental temperature may induce stimulation or reduction of maximum photosynthetic rates depending on the plant functional type [15]. On the other hand, higher CO_2_ concentrations stimulate A_net_ by increasing CO_2_ substrate availability for Rubisco and suppressing photorespiration [11,16]. Long-term exposure to elevated CO_2_ can decrease this photosynthetic stimulation via declines in stomatal conductance or a build-up of leaf carbohydrates that lead to the downregulation of photosynthetic capacity [17]. 

Respiration also can be altered by warming [18,19,20,21]. In contrast to photosynthesis, respiration tends to acclimate to increases in growth temperature to a similar degree in species from different biomes [22,23], where lower respiration rates occur in plants grown at higher temperatures compared to control plants when measured at a common temperature [24]. In the short term, thermal acclimation can often affect the sensitivity of respiration (type I acclimation), manifested through a change in the Q_10_ (the increase in respiration for a 10 °C increase in leaf temperature), reflecting limitations by substrate availability and/or the activity of several enzymes in response to the instantaneous increase in temperature [22,25,26]. Long-term exposure to warmer temperatures usually leads to type II acclimation, where respiration dynamically adjusts to changes in the growth environment [24]. This acclimation affects the overall respiratory capacity, involving changes in the abundance, structure, and/or protein composition of mitochondria [22,24,26,27]. 

Elevated atmospheric CO_2_ concentrations can also influence respiration both in the short and long term; however, the extent of its impact is not yet fully understood. Rapid changes in CO_2_ concentration may have direct, but reversible, effects on respiration [28,29] by decreasing the activity of several enzymes [29,30,31,32,33]. In some studies, prolonged exposure to CO_2_ enrichment decreases respiration rates [19,34,35,36], while in others it leads to elevated respiration rates [37,38]. Griffin et al. (2001) reported that elevated CO_2_ produced significant structural changes, such that increased respiration correlated with an increased number of mitochondria per cell. 

In terms of carbon balance, the ratio of respiration/photosynthesis (R/A) at a certain growth temperature is often constant, even in plants experiencing contrasting growth temperatures ([10] and citations therein). Thus, it is often expected that warming leads to coordinated acclimation of both respiration and photosynthesis to maintain a homeostatic carbon balance [11]. However, elevated CO_2_ affect photosynthesis more strongly than respiration, leading to alterations in the carbon balance of some species [39]. Long-term exposure to elevated CO_2_ will suppress photosynthesis, with potential detrimental consequences on carbon gain [40]; however, downregulation of respiration at higher CO_2_ conditions could reduce this impact. Although some reports affirm that interspecific variation in thermal acclimation of dark respiration is more important than acclimation of respiration to CO_2_ enrichment [18,21,33], the impact of elevated CO_2_ on respiration at a physiological level is still poorly understood. Since elevated CO_2_ seems to have a greater impact on photosynthesis and warming has a stronger impact on respiration, plant carbon dynamics of future vegetation will depend on plant responses to the combination of elevated CO_2_ and warming. In addition, many of the impacts of increased CO_2_ on plant metabolism are offset by increasing temperatures, so these global change factors must be assessed together to build a realistic picture of how a changing climate will impact plants [41]. 

It is important to evaluate the ability of photosynthesis and respiration to acclimate to climate change factors in species from colder habitats, such as Antarctica. In these habitats, photosynthesis is frequently limited (e.g., by low temperatures and highly variable irradiance), and thermal acclimation of respiration and photosynthesis may thus be required to ensure the maintenance of a positive plant carbon balance. The Antarctic Peninsula has experienced one of the most rapid increases in temperature on Earth, and this warming is projected to continue [42,43,44]. In addition, atmospheric CO_2_ concentrations at the South Pole are higher today than they have been in the last 800 years, having surpassed 400 ppm in 2016 [3,45,46]. *Colobanthus quitensis* (Kunth) Bartl. (Carophyllaceae) and *Deschampsia antarctica* Desv. (Poaceae) are the only two vascular plants species that have naturally colonized the Antarctic Peninsula. Their distribution is strongly influenced by climate [47], and local population increases due to climate warming have been documented [48,49]. Bui (2016) evaluated the combined effect of elevated CO_2_ and diurnal warming on photosynthesis of these Antarctic species, suggesting that high CO_2_ could increase photosynthesis at temperatures close to the photosynthetic thermal optimum in *D. antactica*, but not in *C. quitensis*. In contrast, in situ diurnal warming increased photosynthesis in *C. quitensis*, while photosynthesis in *D. antarctica* showed no response to warming [50]. In a previous study, we found that nocturnal warming improves the carbon balance of these two Antarctic species through different mechanisms: respiratory acclimation in *C. quitensis* and increases of maximum light-saturated net CO_2_ assimilation rates (A_sat_) in *D. antarctica* [51]. Although A_sat_ was not significantly increased by nocturnal warming in *C. quitensis*, the higher degree of respiratory thermal acclimation allowed this species to increase its carbon balance under nocturnal warming. However, the combined effects of nocturnal warming with CO_2_ enrichment on foliar carbon balance have not been yet assessed in these Antarctic species. 

In the present study, we examined the extent to which elevated CO_2_ may alter carbon balance in *C. quitensis* and *D. antarctica* when exposed to nocturnal warming. We hypothesized that long-term exposure to concurrent elevated CO_2_ conditions, and nocturnal warming would lead to decreased photosynthesis in both Antarctic species. However, the impact of this decline in photosynthesis on carbon balance would depend on the extent of thermal acclimation of respiration to nocturnal warming, which was expected to be greater in *C. quitensis* than *D. antarctica*. To determine the mechanism underlying respiration acclimation, the main substrates for respiration (total soluble sugars (TSS) and starch) were evaluated. Additionally, the relative concentration of two respiratory metabolism enzymes (Phosphoenol-pyruvate carboxylase (PEPc) and cytochrome oxidase (COXII)) important in replenishing oxalacetate in the tricarboxylic acid cycle and reducing oxygen to water and coupling ATP production in the electron transport chain, respectively. The number and size of mitochondria were also evaluated in leaves from across the CO_2_ and temperature treatments to determine if long-term thermal acclimation of respiration involved changes in the abundance or structure of mitochondria.

## 2. Results

### 2.1. Gas Exchange and Carbon Balance

**Figure 1:**
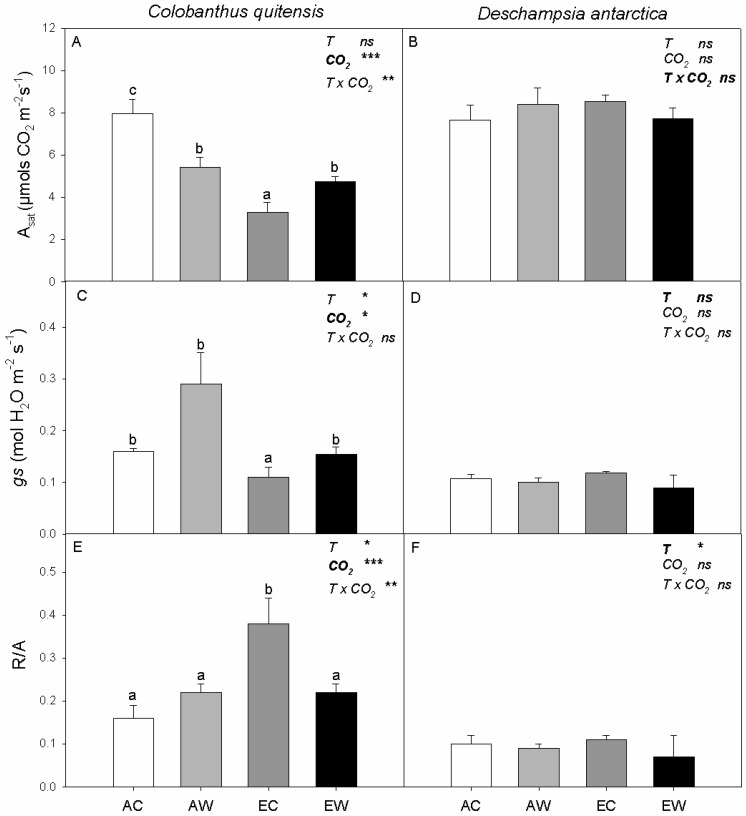
Net CO_2_ assimilation rate measured at saturating light and 400 ppm of CO_2_ (A_sat_), stomatal conductance (*gs*) and foliar leaf carbon balance (R/A) for *C. quitensis* (**A**,**C**,**E**) and *D. antarctica* (**B**,**D**,**F**). Treatments correspond to AC (ambient CO_2_, control thermoperiod; white bar empty), AW (ambient CO_2_, warming thermoperiod; white bar hashed), EC (elevated CO_2_, control thermoperiod; grey bar), and EW (elevated CO_2_, warming thermoperiod; grey bar hashed). Values are means ± SEM (*n* = 5). For each graph, the effect of thermoperiod (T), CO_2_ environment (CO_2_), and the interaction of thermoperiod and CO_2_ (T x CO_2_), ns indicates no significance difference, * indicates *p* ≤ 0.05, ** indicates *p* ≤ 0.01, and *** indicates *p* ≤ 0.001. The factor with the largest effect size is indicated in bold.

In *C. quitensis,* there was a CO_2_ x warming interaction effect for A_sat_. The A_sat_ was 30% lower in AW than AC plants, while A_sat_ increased in EW plants compared to EC plants (Figure 1A). In contrast, neither elevated CO_2_ nor warming altered A_sat_ in *D. antarctica* (Figure 1B). The stomatal conductance (*gs*) was increased by nocturnal warming and decreased in elevated CO_2_ in *C. quitensis* (Table 1; Figure 1C). In contrast, neither elevated CO_2_ nor warming altered *gs* were found in *D. antarctica* (Figure 1D).

**Figure 2:**
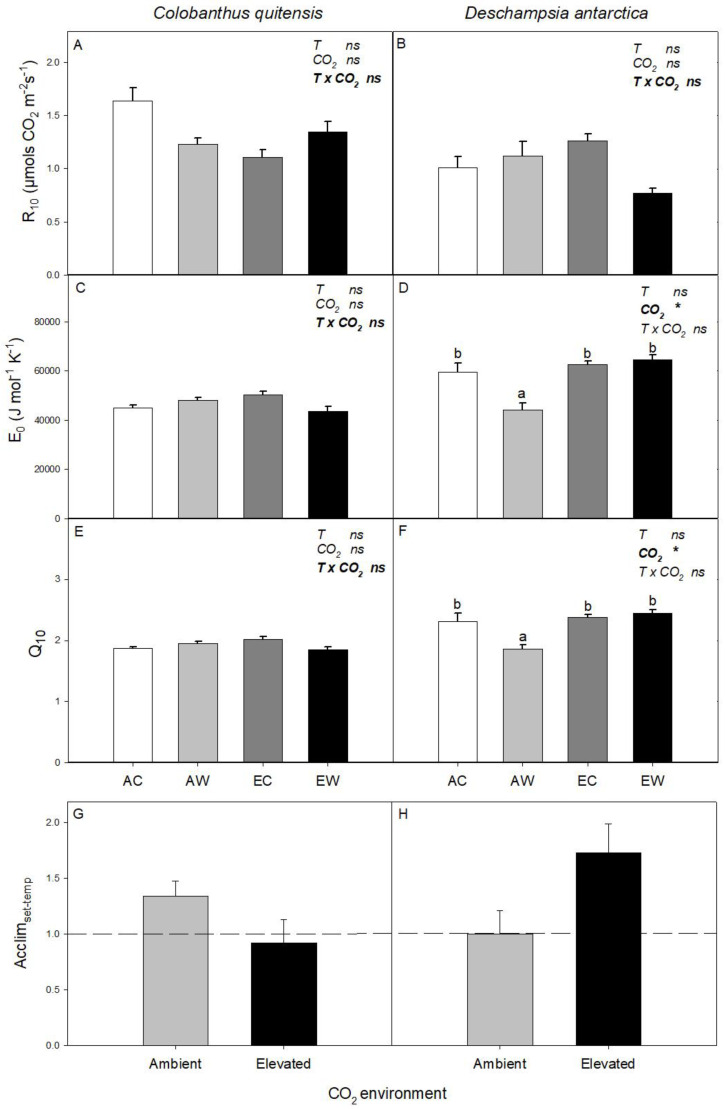
Sensitivity parameters of dark respiration calculated using the Arrhenius equation for both Antarctic species. R_10_ is respiration at 10°C, E_0_ is a modelled parameter related to the energy of activation, and Q_10_ denotes the relative change in respiration with a 10°C change for *C. quitensis* (**A**,**C**,**E**) and *D. antarctica* (**B**,**D**,**F**). Treatments correspond to AC (ambient CO_2_, control thermoperiod; white bar empty), AW (ambient CO_2_, warming thermoperiod; white bar hashed), EC (elevated CO_2_, control thermoperiod; grey bar), and EW (elevated CO_2_, warming thermoperiod; grey bar hashed). The acclimation degree was calculated as Acclim_set-temp_ = R_control_/R_warming_ at ambient and elevated CO_2_ for *C. quitensis* (**G**) and *D. antarctica* (**H**). Values are means ± SEM (*n* = 5). For each graph, the effect of thermoperiod (T), CO_2_ environment (CO_2_), and the interaction of thermoperiod and CO_2_ (T x CO_2_), ns indicates no significance difference, * indicates *p* ≤ 0.05, ** indicates *p* ≤ 0.01, and *** indicates *p* ≤ 0.001. The factor with the largest effect size is indicated in bold.

In *C. quitensis*, changes in atmospheric CO_2_ concentration and nocturnal warming did not significantly affect R_10_, E_0_, or Q_10_ (Figure 2A,C,E). In *D. antarctica*, R_10_ was not affected by either treatment (Figure 2B), whilst the E_0_ and Q_10_ were significantly higher in plants grown at elevated CO_2_ than at ambient CO_2_ (Figure 2D,F), which was largely driven by low E_0_ and Q_10_ values in the AW treatment. 

The Acclim_set-temp_ for *C. quitensis* was 1.34 ± 0.14 and 0.92 ± 0.21 for ambient and elevated CO_2_-grown plants, respectively. For *D. antarctica*, values of Acclim_set-temp_ were 1.00 ± 0.21 and 1.73 ± 0.26 for ambient and elevated CO_2_, respectively (Figure 2G,H). There was no significant effect of elevated CO_2_ on Acclim_set-temp_ for either species (*p* = 0.14 and *p* = 0.06 for *C. quitensis* and *D. antarctica*, respectively).

For foliar carbon balance, the ratio of R/A was not affected by night temperature in the ambient CO_2_ grown *C. quitensis* but was almost 50% lower in EW plants than in EC plants. Additionally, R/A was higher in the elevated CO_2_ treatment than in the ambient CO_2_ treatment for *C. quitensis* (Figure 1E), which was largely driven by the high R/A in the EC individuals. In contrast, the R/A ratio showed a small, but significant, decrease in the warming treatments in *D. antarctica* (Figure 1F) but was not affected by the CO_2_ treatments.

### 2.2. Non-Structural Carbohydrates, Relative Abundances of PEPc and COX-II

**Figure 3:**
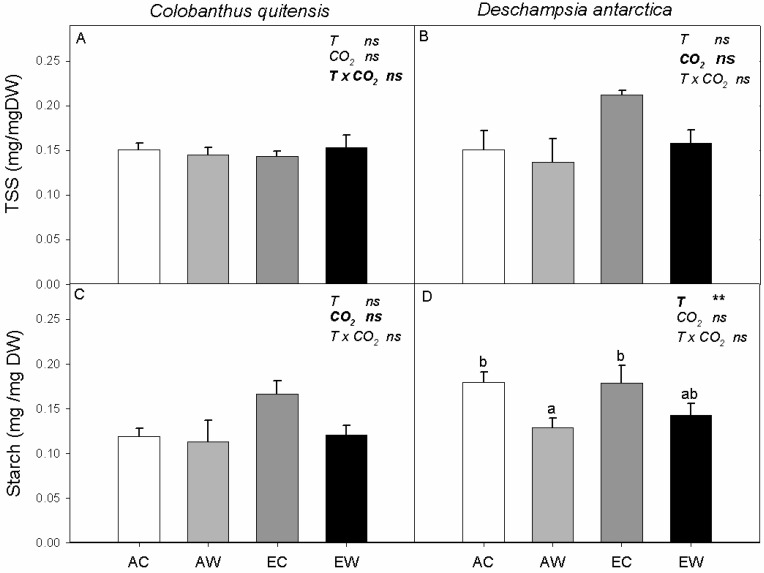
Total soluble sugars (TSS) and starch for *C. quitensis* (**A**,**C**) and *D. antarctica* (**B**,**D**). Treatments correspond to AC (ambient CO_2_, control thermoperiod; white bar empty), AW (ambient CO_2_, warming thermoperiod; white bar hashed), EC (elevated CO_2_, control thermoperiod; grey bar), and EW (elevated CO_2_, warming thermoperiod; grey bar hashed). Values are means ± SEM (*n* = 5). For each graph, the effect of thermoperiod (T), CO_2_ environment (CO_2_), and the interaction of thermoperiod and CO_2_ (T x CO_2_), with ns indicates no significance difference, * indicates *p* ≤ 0.05, ** indicates *p* ≤ 0.01, and *** indicates *p* ≤ 0.001. The factor with the largest effect size is indicated in bold.

Neither total soluble sugar (TSS) nor starch concentrations of *C. quitensis* were affected by the CO_2_ and thermoperiod treatment (Figure 3A,C). However, in *D. antarctica*, while TSS concentrations were not significantly affected by growth treatments, starch concentrations were reduced by nocturnal warming (Figure 3B,D).

**Figure 4:**
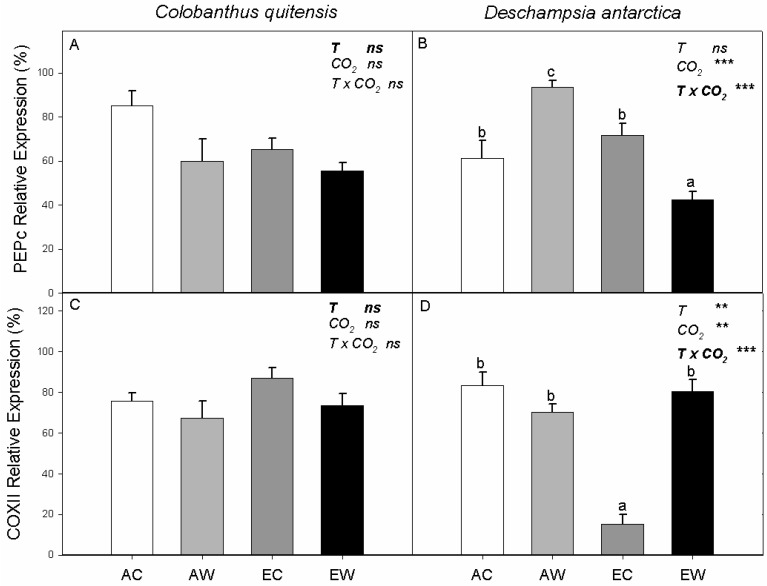
Relative abundance of phosphoenol pyruvate carboxylase (PEPc) and cytochrome oxidase (COX-II) proteins for *C. quitensis* (**A**,**C**) and *D. antarctica* (**B**,**D**). Treatments correspond to AC (ambient CO_2_, control thermoperiod; white bar empty), AW (ambient CO_2_, warming thermoperiod; white bar hashed), EC (elevated CO_2_, control thermoperiod; grey bar), and EW (elevated CO_2_, warming thermoperiod; grey bar hashed). Values are means ± SEM (*n* = 5). For each graph, the effect of thermoperiod (T), CO_2_ environment (CO_2_), and the interaction of thermoperiod and CO_2_ (T x CO_2_), with ns indicates no significance difference, * indicates *p* ≤ 0.05, ** indicates *p* ≤ 0.01, and *** indicates *p* ≤ 0.001. The factor with the largest effect size is indicated in bold.

Neither the relative abundance of PEPc nor that of COX-II were significantly affected by the treatments in *C. quitensis* (Figure 4A,C). In *D. antarctica*, elevated growth CO_2_ concentrations suppressed the relative expression of PEPc, with warming increasing PEPc relative abundance in ambient CO_2_-grown plants but reducing PEPc relative abundance in plants grown at elevated CO_2_ (Figure 4B). There was also an interactive effect of growth CO_2_ and nocturnal temperature on COX-II relative abundance in *D. antarctica*, resulting from a strong suppression of COX-II levels in EC plants compared to the other three treatments (Figure 4D). 

### 2.3. Mitochondrial Traits

**Figure 5:**
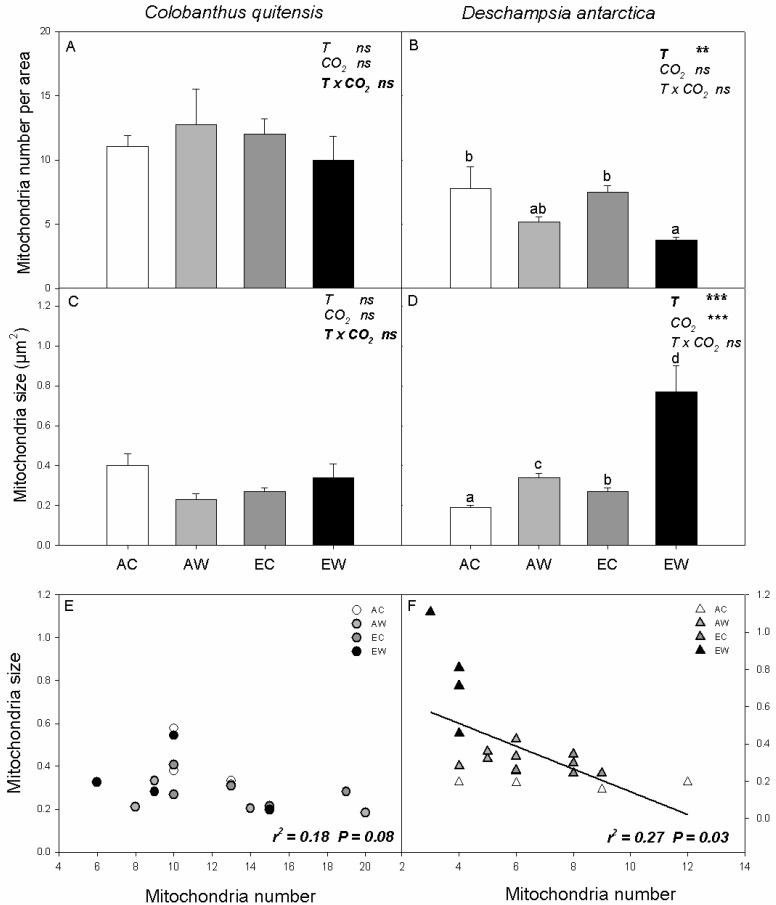
Leaf mitochondria structural changes in number, and size of mitochondria in a determined area of 171.8 µm^2^ and their correlation for *C. quitensis* (**A**,**C**,**E**) and *D. antarctica* (**B**,**D**,**F**) grown at AC (ambient CO_2_, control thermoperiod; white bar empty), AW (ambient CO_2_, warming thermoperiod; white bar hashed), EC (elevated CO_2_, control thermoperiod; grey bar), and EW (elevated CO_2_, warming thermoperiod; grey bar hashed). Values are means ± SEM (*n* = 5). For each graph, the effect of thermoperiod (T), CO_2_ environment (CO_2_), and the interaction of thermoperiod and CO_2_ (T x CO_2_), with ns indicates no significance difference, * indicates *p* ≤ 0.05, ** indicates *p* ≤ 0.01, and *** indicates *p* ≤ 0.001. The factor with the largest effect size is indicated in bold.

Neither the number nor the size of mitochondria in *C. quitensis* was affected by CO_2_ or warming (Figure 5A,C). In *D. antarctica,* nocturnal warming reduced the number of mitochondria, but increased mitochondrial size, especially in EW plants (Figure 5B,D). While there was no correlation between mitochondrial size and number in *C. quitensis* (Figure 5E), *D. antarctica* showed a tradeoff between mitochondrial size and number, whereby smaller numbers of mitochondria were correlated with larger mitochondrial size (Figure 5F). 

**Figure 6:**
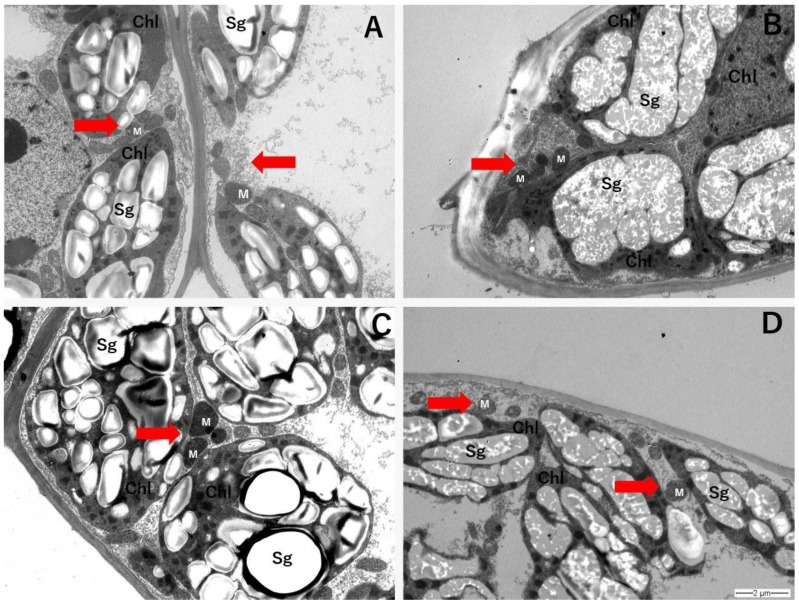
Mitochondria (M; red arrows), chloroplasts (Chl), and starch granules (Sg) from leaf mesophyll of *C. quitensis* exposed to AC (ambient CO_2_, control thermoperiod; (**A**), AW (ambient CO_2_, warming thermoperiod; (**B**), EC (elevated CO_2_, control thermoperiod; (**C**), and EW (elevated CO_2_, warming thermoperiod; (**D**) Microscope magnification = 6000X.

**Figure 7:**
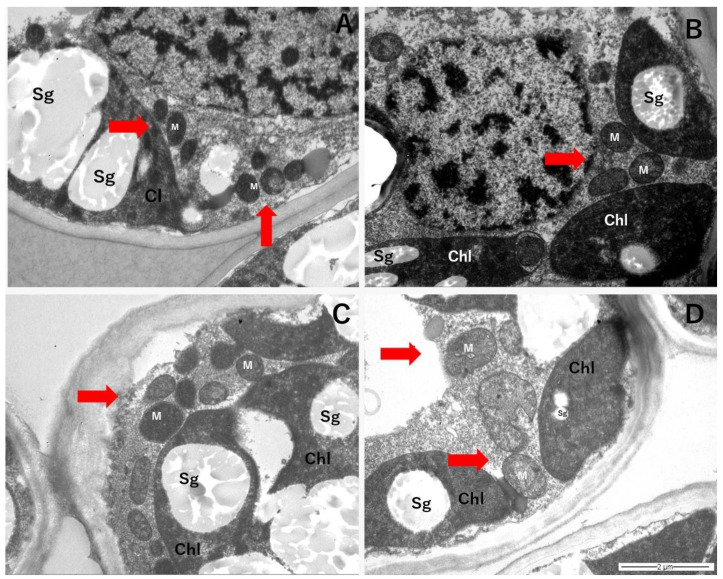
Mitochondria (M; red arrows), chloroplasts (Chl), and starch granules (Sg) from leaf mesophyll of *D. antarctica* exposed to AC (ambient CO_2_, control thermoperiod; (**A**), AW (ambient CO_2_, warming thermoperiod; (**B**), EC (elevated CO_2_, control thermoperiod; (**C**), and EW (elevated CO_2_, warming thermoperiod; (**D**). Microscope magnification = 11,500X.

For *C. quitensis,* mitochondria from each growth condition appeared to maintain a similar shape, and large starch granules were visible inside the chloroplasts in all samples observed (Figure 6). For *D. antarctica*, despite changes in the mitochondrial size under warming treatments, there were no obvious changes in the shape of mitochondria between different growing conditions (Figure 7), though neither mitochondrial shape nor the presence of starch granules in the mitochondria could be assessed quantitatively.

## 3. Discussion

In this study, we evaluated the effects of elevated CO_2_ and nocturnal warming on gas exchange and related the biochemistry and anatomy of Antarctic species in order to determine the extent to which elevated CO_2_ may alter the carbon balance in *C. quitensis* and *D. antarctica* under nocturnal warming conditions. We found that these two species showed different acclimation strategies in the face of combined elevated CO_2_ and warm night temperatures. In *C. quitensis*, the downregulation of A_sat_ in long-term EC grown plants suggests a strong metabolic adjustment at chloroplast level, which could involve Rubisco reductions. In this species, the short-term sensitivity of respiration and the relative abundances of PEPc and COXII indicate a lack of respiratory response at most of the conditions tested. Indeed, the only respiratory parameter that showed any response to warming or elevated CO_2_ in this species was Acclim_set-temp_, which suggests some degree of thermal acclimation at the ambient CO_2_-acclimated plants. Reduced photosynthesis measured at 400 ppm of CO_2_ and limited respiratory acclimation can lead to an increased ratio of R/A in *C. quitensis* when grown in elevated CO_2_. However, we did not evaluate A_sat_ at 750 ppm for any species; thus, it was not possible to assess whether photosynthesis acclimation at elevated CO_2_ occurred. Contrary to *C. quitensis*, in *D. antarctica*, the A_sat_ and *gs* did not change at any CO_2_ or warming treatments. Moreover, for both species, the high amounts of starch granules in leaf tissues suggest high photosynthetic rates in plants growth at elevated CO_2_. E_0_ and Q_10_ were higher in plants exposed to elevated CO_2_ levels than in those under ambient CO_2_ conditions, leading to type I acclimation, mediated by changes in the relative abundance of PEPc and COXII, suggesting reductions in TCA cycle intermediates and variations in ATP production, respectively. The combined effect of elevated CO_2_ and nocturnal warming resulted in a smaller quantity of mitochondria, but they were of the largest size, reflecting a high capacity of type II acclimation in this species.

### 3.1. Elevated CO_2_ and Nocturnal Warming Differentially Affected the Photosynthetic Performance of the Two Antarctic Species

Most plants tend to show a downregulation of photosynthesis when acclimated to long-term high CO_2_ conditions [11,52,53]. This CO_2_-induced downregulation in photosynthesis could be linked to shifts in the CO_2_ supply via changes in *gs* [54]. It could also be attributed to a sugar feedback mechanism, through which excessive photosynthate concentrations in chloroplasts are thought to repress the transcription of Rubisco [53]. In *C. quitensis,* elevated CO_2_-induced reduction in stomatal conductance resulted in a significant decrease in A_sat_ measured at 400 ppm. Previous studies have affirmed that *gs* plays an important role in carbon gain in Antarctic species, offsetting the diffusive limitation imposed by the extremely low mesophyll conductance in both species [50]. The downregulation of A_sat_ in EC grown plants suggests a strong metabolic adjustment at the chloroplast level, involving Rubisco reductions. Long-term exposure to CO_2_ enrichment has been associated to decreased Rubisco protein and nitrogen reallocation to more limiting process [55] or even as a source of amino acids, because nitrate assimilation also can become inhibited by elevated growth CO_2_ [56]. Moreover, photosynthesis in the elevated CO_2_-grown plants were not evaluated at 750 ppm; thus, it was not possible to assess whether photosynthesis acclimation at elevated CO_2_ occurred. Despite elevated CO_2_ levels in *D. antarctica,* they had no effect on stomatal conductance and carbon assimilation at ambient CO_2_; this does not mean that A_sat_ evaluated at 750 ppm could be higher than at 400 ppm. In addition, in both species, the high amount of visible starch by electron microscopy (Figure 6C and Figure 7C) suggests high photosynthetic rates in plants grown at elevated CO_2_. Further experiments would eventually include the evaluation of acclimation of A_sat_ and changes of foliar nitrogen at the new growth CO_2_ environment.

Nocturnal warming may induce overconsumption (or accumulation) of carbohydrates, resulting in elevated (or decreased) rates of photosynthesis, respectively [57,58]. A photosynthetic downregulation have been associated with decreases in stomatal conductance, production of reactive oxygen species, and the deactivation of several key chloroplast stromal enzymes at night [58,59,60,61]. In accordance with previous reports of experimental diurnal warming, here, both Antarctic plants showed contrasting responses to nocturnal warming [50,62]. Although the warming conditions of both CO_2_ environments reduced A_sat_ in *C. quitensis*, the interaction of both factors (EW) largely offset the downregulation of photosynthesis at elevated CO_2_ in plants measured at 400 ppm of CO_2_. The positive effect of this interaction could be related to specific adjustments in the leaves of this species at the anatomical level [50]. Despite this, in *C. quitensis,* warming-induced reductions in A_sat_ that were not related to *gs* and could be associated with enzymatic or metabolic changes in response to increased night-time temperatures [58,63]. For *D. antarctica,* responses to nocturnal warming were similar to previous reports on daily warming, showing no changes in photosynthesis due to warming, at least at suboptimal temperatures [50,64]. TSS levels of both Antarctic plants were apparently unaffected by increased CO_2_ levels or warming, suggesting either there were changes in the turnover rate of these pools or increased carbohydrate export. Further studies examining photosynthetic enzymes, metabolic intermediates, and carbohydrate translocation dynamics from leaves to roots are necessary to elucidate the effect of nocturnal warming on the photosynthesis of Antarctic plants. Most studies have focused on evaluating the interaction between higher daytime temperatures and CO_2_ enrichment [11,21,38,39,65,66], while few have evaluated the interaction with increased night-time temperatures. Turnbull et al. (2004) reported that the increase in photosynthesis induced by warmer night-time temperatures was not affected by elevated CO_2_ [67]; however, Cheng et al. (2009) reported that increased photosynthesis at elevated CO_2_ levels was offset by high night-time temperatures [68]. Here, we found that nocturnal warming could alleviate the downregulation on A_sat_, particularly for *C. quitensis*. Considering that the two evaluated Antarctic species showed interspecific responses of A_sat_ to elevated CO_2_ at 400 ppm of CO_2_ and nocturnal warming, we hypothesized that the effect of warming depended on the CO_2_ environment, highlighting the need for factorial experiments that expose plants to multiple global change factors, especially considering species from extreme environments.

### 3.2. Dark Respiration Showed Differential Sensitivity and Thermal Acclimation to Elevated CO_2_ and Nocturnal Warming 

In agreement with Sanhueza et al. (2019), nocturnal warming altered the thermal sensitivity of respiration in *D. antarctica*, while respiration was unaffected by warming in *C. quitensis*. According to the mechanisms underlying respiratory acclimation type I, proposed by Atkin and Tjoelker (2003), substrate availability determines changes in Q_10_ [69,70]. Thus, in *D. antarctica* exposed to nocturnal warming, a decrease in Q_10_ was attributed to a greater depletion of starch to support respiration in leaves or sink organs [26]. The enzyme PEPc, which catalyzes the conversion of phosphoenolpyruvate to oxaloacetate in the cytosol and is subsequently reduced to malate that is then utilized in the TCA cycle [29], was affected differently by warming depending on the CO_2_ growth environment. Thus, a significant decrease in the relative expression of PEPc under EW could reflect a reduction in the entrance of pyruvate or malate to the TCA cycle, which is because of intermediates’ reduction, which may decrease decarboxylation reactions. This response could explain the slight (yet non-significant) reduction of R_10_ in this species. Noguchi et al. (2018) reported that both the ratio of intermediates and the maximal activity of enzymes involved in the TCA cycle changed at elevated CO_2_ levels; they also related these changes to an increased amino acid production [71].

Considering that responses related to carbon release at the TCA level do not necessarily correspond to what is occurring at the electron transport chain level, the quite different responses at the ETC scale suggest that the respiratory metabolism of *D. antarctica* has a high plasticity, mainly at elevated CO_2_ growth. Variations in the relative COX II content, evaluated as a proxy for the cytochrome oxidase pathway (COP), suggest changes at the energy production level (ATP). However, any negative effect of high CO_2_ (e.g., a significant decrease of COX II at EC) seems to be offset under nocturnal warming when both factors occur simultaneously (EW). This is in accordance with several studies where plants have shown opposite responses to increased CO_2_ and warming, even when comparing species from the same functional group [66,67,69,72].

We hypothesized that elevated CO_2_ accompanied by nocturnal warming would enhance the thermal acclimation of respiration in *C. quitensis* more than in *D. antarctica*. However, we found little evidence for respiratory acclimation to warming under elevated CO_2_ in *C. quitensis*, contrary to *D. antarctica*, which showed a high degree of thermal acclimation. The Acclim_set-temp_ values reported here are higher than those reported for forbs and graminoids inhabiting polar regions, though they are similar to previously reported values for Antarctic plants and other plant species under warmer growth conditions [51,70,73,74]. Respiratory acclimation to warmer temperatures generally results in lower respiration rates, which have been associated with smaller mitochondria [75], although few reports have related respiration rates with mitochondrial structural changes. Griffin et al. (2001) reported that elevated CO_2_ reduced respiration rates and mitochondrial numbers, without affecting mitochondrial size [37]. For *D. antarctica,* respiratory acclimation at elevated CO_2_ under warming resulted in a tradeoff between mitochondrial size and number (Figure 5F). Increases in mitochondria size at EW may reflect the production of new respiratory components mainly at the mETC. The effect of elevated CO_2_ on components of mETC in *Nicotiana tabacum* doubled the amount of alternative oxidase (AOX) protein in leaves [76] to maintain both the carbon and energy balance in photosynthetic tissues during growth under these conditions. For *D. antarctica,* exposure to higher temperatures has been reported to increase AOX activity in leaves as a consequence of higher metabolic activity [77]. The AOX respiratory pathway could play a role in the reduction of the reactive oxygen of species and even aid plants in coping with excessive energy in chloroplasts, thus avoiding over-reduction [63,78]. Consequently, in this species, the mechanisms underlying respiratory acclimation at elevated CO_2_ proved to be highly dynamic, comprising complex physiological, biochemical, and molecular adjustments. Future experiments must evaluate the regulation of the two respiratory pathways under warming and elevated CO_2_ in Antarctic plants in order to further understand the effect of climate change on respiration and growth.

### 3.3. The Determining Factor to Maintain the Carbon Balance in Antarctic Species Appears to Be the Maintenance of the Photosynthetic Rate

A reduction in photosynthetic performance due to environmental changes could be detrimental for the maintenance of carbon gain in any species. Furthermore, if this condition is maintained over time, it could reduce growth and survival [79]. In *C. quitensis,* the strong decline in A_sat_ at 400 ppm under elevated CO_2_ levels in addition to the lack of respiratory response at this condition significantly increased the ratio of photosynthesis to respiration, which eventually could harm the foliar carbon balance in this species. Moreover, the evaluation of assimilation rates at 750 ppm of CO_2_ could better explain the effect of CO_2_ on carbon gain under a new growth condition. Despite this, greater plasticity at the mitochondrial respiratory level can also give an advantage in terms of an adequate maintenance on carbon balance. In this way, in *C. quitensis*, most of the physiological parameters evaluated across this study suggest a low capacity for respiration acclimation to elevated CO_2_ and nocturnal warming in this species. In contrast, in *D. antarctica*, the capacity to maintain high photosynthetic rates at warmer nights and elevated CO_2_ seemed to respond to the modification of traits related to mitochondrial respiration, thus contributing to maintain the leaf carbon balance. Thus, the high capacity for morphological and physiological adjustments of *D. antactica* seems to be an important trait helping to tolerate environmental changes and contributing to increase its ability to successfully colonize and spread throughout the Antarctic Peninsula. 

## 4. Materials and Methods

### 4.1. Plant Material

*Colobanthus quitensis* (Kunth) Bartl. (Antarctic pearlwort) and *Deschampsia antarctica* Desv. (Antarctic hairgrass) were collected near H. Arctowski Polar Antarctic Station on King George Island (62°09′ S, 58°28′ W), corresponding to an intermediate vegetation zone [50]. Plants of both species (24 individuals per species) were wrapped in moist paper, sealed in Ziploc bags, and transported in Styrofoam boxes kept cool with ice packs, before being transported to the Biotron Centre for Experimental Climate Change Research at the University of Western Ontario, London, ON, Canada. Individuals of each species were planted into 10 cm diameter pots (0.5 L) with a potting medium of black loam/peat moss/vermiculite (3/1/1, *v*/*v*/*v*). Plants were kept in a walk-in growth chamber (Environmental Growth Chambers, Chagrin Falls, OH, USA) at 10 °C and 300 μmol photons m^−2^ s^−1^, with an 18/6 h light/dark cycle and ~60% relative humidity to minimize any stress incurred during transport. Two weeks later, plants were moved into the combined elevated CO_2_ and nocturnal warming experiment.

### 4.2. Experimental Design 

The experiment was a full-factorial design with two different CO_2_ environments (ambient, A, 400 ppm CO_2_; elevated, E, 750 ppm CO_2_) and two nocturnal thermoperiods (control, C, 8/5°C; warming, W, 8/8 °C). The different treatments applied will be reported as AC (ambient CO_2_, control thermoperiod), AW (ambient CO_2_, nocturnal warming), EC (elevated CO_2_, control thermoperiod), and EW (elevated CO_2_, nocturnal warming). The control temperatures represent values close to the maximum air temperatures registered during the Maritime Antarctic summer [50]. Each experimental condition was achieved in an independent walk-in growth chamber under the same light intensity and photoperiod as described above. In each chamber, plants were watered twice a week, fertilized with half-strength Hoagland’s solution at the beginning of the experiment, and maintained for 21 days, corresponding to time period in which both Antarctic species reach a high capacity of acclimation [47,50,80,81]. Potential chamber and edge effects were minimized by rotating the plants among the chambers every four days.

### 4.3. Gas Exchange 

Gas exchange measurements were performed using a portable photosynthesis system (Li-6400XT, LI-COR Inc., Lincoln, NE, USA) on a set of leaves inside a 2 cm^2^ cuvette (from either a branch of *C. quitensis* or a tiller of *D. antarctica*), to maximize leaf cover of the cuvette area while avoiding leaf overlap. When the leaf area was smaller than the cuvette area, the actual leaf was photographed and analyzed using ImageJ software (US National Institutes of Health, Bestheda, MD, USA).

Temperature response curves of leaf dark respiration were measured at 5, 10, 15, 20, 25, and 30 °C, under cuvette conditions of 400 ppm CO_2_, ~60% relative humidity, and an irradiance of 0 µmol photons m^−2^ s^−1^ between 9:00 and 18:00 on five replicates per species per treatment. Leaves were exposed to at least 30 min of darkness before the first measurement was made. The temperature response curves were analyzed by fitting each leaf temperature-respiration measurement to a modified Arrhenius equation [51,57,82]

R = R_10_exp [(E_0_/*g*) (1/T_10_ − 1/T_i_)], (1)

From the fitted equations for each replicate plant, we obtained R_10_ (the dark respiration rate at 10 °C, in µmol m^−2^ s^−1^) and E_0_, which is equivalent to the overall activation energy of the process (in Jmol^−1^K^−1^) and describes the temperature sensitivity of respiration [83]. The Q_10_, corresponding to the temperature sensitivity of respiration, was calculated as:

Q_10_ = (R/R_ref_) exp [10/T − T_ref_]. (2)

This approach allows for the calculation of Q_10_ for temperature intervals that derivate from 10 °C, where T_ref_ is the low reference temperature and R_ref_ is the respiration rate at this reference temperature [83].

Maximum net CO_2_ assimilation rates at saturating light (A_sat_) were measured on the same five plants that were measured for respiration from each treatment. The A_sat_ was measured at saturating irradiance (1000 µmol photons m^−2^s^−1^) at 10 °C, 400 ppm CO_2_ and ~60% relative humidity. Estimates of leaf carbon balance (R/A) were obtained from the ratio of R_10_ to A_sat_. 

### 4.4. Quantification of Thermal Acclimation of Respiration

The set temperature method was used to quantify the degree of respiratory thermal acclimation [22,51], as per [84]:

Acclim_set-temp_ = R_control_/R_warming_, (3)

where Acclim_set-temp_ indicates the strength of acclimation, R_control_ is R_10_ from the control night temperature plants, and R_warming_ is R_10_ from the warm-acclimated plants. Two values of Acclim_set-temp_ were obtained for each species, corresponding to thermal acclimation at either an ambient CO_2_ or an elevated CO_2_ environment. An Acclim_set-temp_ of >1 indicates thermal acclimation, while an Acclim_set-temp_ of <1 indicates no thermal acclimation occurred in warm-grown plants. 

### 4.5. Biochemical Analyses 

Leaf samples were collected from five plants per species per treatment for biochemical and anatomical analyses after measurements of gas exchange. Total soluble sugar (TSS: combined glucose, fructose, and sucrose concentrations) and starch concentrations were evaluated following the method of Marquis et al. (1997), using 15 mg of lyophilized leaf tissue [85]. Total soluble sugars were extracted with a methanol/chloroform/water (12/5/3, *v*/*v*/*v*) solution separated from nonpolar pigments and lipids according to Dickinson (1979) and determined by colorimetry using phenol 2% and sulfuric acid, and a measuring absorbance of 490 nm [86,87]. Starch from the insoluble fraction was hydrolyzed to glucose overnight using a sodium acetate buffer and amyloglucosidase (Sigma-Aldrich 10115, St. Louis, MO, USA) at 45 °C and then measured with a phenol-sulfuric acid reaction [85].

The relative protein content of phosphoenolpyruvate carboxylase (PEPc) and cytochrome oxidase (COX-II) were evaluated from the same leaf samples as the carbohydrates. Total protein extractions were performed following the method of Yamori and Von Cammerer (2009) [88]. Then, 100 g of lyophilized tissue was mixed with buffer with 50 mM HEPES-KOH (pH = 7.8), 10 mM MgCl2, 1 mM EDTA, 5 mM DTT, and 0.1% Triton X-100 (*v*/*v*). The extracts were centrifuged at 8000 rpm for 1 min at 4 °C. A total of 230 µL of the supernatant was taken and 70 μL of 10% SDS was added by heating at 65 °C for 10 min. Next, 45 μL of the extract was added to 15 μL of 4X sample buffer containing 250 mM Tris-HCl (pH = 6.8), 40% glycerol, 8% SDS, 0.2% bromophenol, and 200 mM DDT. The extract was then heated at 100 °C for 5 min and centrifuged at 8000 rpm for 1 min. Subsequently the extracts were incubated in a fridge until SDS-PAGE electrophoresis. Leaf extracts were separated by sodium dodecyl sulphate-polyacrilamide gel electrophoresis using a 4–12% gradient polyacrylamide gels and then transferred to a nitrocellulose membrane at 100 v for 1 h and visualized using Ponceau Red staining (Merck). Subsequently, the total proteins were immunolocalized with PEPc and COXII antibodies (Goat Anti-Rabbit HRP conjugated, Vännäs, SWEDEN) at a concentration of 1:1000. The proteins were detected by chemiluminescence (ECL) (Pierce, Rockford, IL, USA) on an X-ray film (Fuji, Tokyo, Japan). The densitometric chemiluminescent bands produced on the X-ray plates were quantified with the software Image J (v1.4 Wayne Rasband, National Institute of Health, Kensington, MD, USA). Results were expressed as a percentage of the maximum protein level determined. A standard sample of each species was run on each blot and all samples are reported normalized to this standard. Protein abundance for each sample was then divided by the mean abundance of all analyzed samples for each protein and species to equalize the distribution for both proteins [82].

### 4.6. Transmission Electron Microscopy of Leaf Mesophyll

Fresh leaf samples were collected from fully expanded leaves of five *C. quitensis* and *D. antarctica* plants from each treatment after the gas exchange measurements. Leaf sections of 1 mm^2^ were fixed in 4% glutaraldehyde and post-fixed with 1% osmium tetroxide. Leaves were analyzed with a transmission electron microscope (TEM Jeol, JEM1200 EXII) at a voltage intensity of 60 kV. The photomicrographs were analyzed using Image J software. The number of mitochondria per microscope field and mitochondrial size (µm) from each sample were determined at 6000X and 11,500X for *C. quitensis* and *D. antarctica*, respectively.

### 4.7. Statistical Analyses 

Analysis of variance (ANOVA) tests were used to assess statistical differences between the temperature and CO_2_ treatments. Most of the evaluated parameters were analyzed by two-way ANOVA using CO_2_ concentrations, nocturnal thermoperiods, and their interaction as factors. Additionally, for each factor, *p* values and the effect size of each factor were calculated (Table 1). Thermal acclimation differences (e.g., Acclim_set-temp_) were evaluated with a one-way ANOVA, using CO_2_ concentration as the factor. When the ANOVA showed significant differences (*p* < 0.05), a post hoc Fisher test was applied to evaluate differences between treatments. Before performing analyses, data were checked for normality and homogeneity of variances. Pearson correlation was used to assess the relation between mitochondrial number and size. All analyses were performed with InfoStat/L (FCA-UNC, Argentina, V 10.0).

## 5. Conclusions

In this study, *C. quitensis* and *D. antarctica* deployed different mechanisms when acclimated to future climate scenarios, including nocturnal warming and elevated CO_2_. Changes in photosynthesis and mitochondrial respiration at new growth conditions are important factors determining the foliar carbon balance in both Antarctic species. Any factor suppressing carbon uptake places the plant carbon balance at risk, which could affect growth and, consequently, survival. In this context, in the face of a future scenario involving increased CO_2_ levels accompanied by nocturnal warming, a lower capacity to maintain photosynthetic performance, and a low capacity of acclimate respiration could be detrimental for *C. quitensis*, while the ability of *D. antarctica* to maintain photosynthesis and mainly adjust its respiratory metabolism could allow this species to continue its successful colonization throughout the Antarctic Peninsula. 

## Figures and Tables

**Table 1:** Results of two-way ANOVA and the size effects (η^2^; Eta squared) of each evaluated factor. The response variables were net CO_2_ assimilation at saturating light and 400 ppm of CO_2_ (A_sat_), stomatal conductance (*gs*), dark respiration at 10 °C (R_10_), activation energy of respiration (E_0_), thermal sensitivity of respiration (Q_10_), thermal acclimation of respiration (Acclim_set-temp_), foliar carbon balance (R/A), total soluble sugar concentrations (TSS), starch concentrations, phosphoenol-pyruvate carboxylase (PEPc) concentrations, cytochrome oxidase (COXII) concentrations, mitochondrial number, and mitochondrial size. Significant effects (*p* < 0.05) are indicated in bold. The η^2^ values were calculated from information in the ANOVA table as η^2^ = Treatment sum of square/ (treatment sum of square + total sum of squares).

***C. quitensis***	***p* Value**			**η^2^**		
**Response Variable**	**T**	**CO_2_**	**T x CO_2_**	**T**	**CO_2_**	**T x CO_2_**
A_sat_	0.30	**<0.001**	**0.00**	0.02	0.32	0.21
gs	**0.02**	**0.01**	0.59	0.20	0.24	0.01
R_10_	0.60	0.22	0.06	0.01	0.07	0.16
E_0_	0.49	0.90	0.09	0.03	0.00	0.14
Q_10_	0.51	0.82	0.09	0.02	0.00	0.15
Acclim_set-temp_	-	0.14	-	-	0.20	-
R/A	**0.02**	**<0.001**	**<0.001**	0.12	0.29	0.27
TSS	0.79	0.96	0.41	0.00	0.00	0.04
Starch	0.13	0.10	0.22	0.10	0.12	0.07
PEPc	0.06	0.49	0.39	0.27	0.04	0.06
COXII	0.09	0.16	0.69	0.16	0.11	0.01
Mit. Number	0.58	0.82	0.24	0.02	0.00	0.09
Mit.Size	0.09	0.78	0.05	0.14	0.00	0.18
***D. antarctica***	***p* Value**			**η^2^**		
**Response Variable**	**T**	**CO_2_**	**T x CO_2_**	**T**	**CO_2_**	**T x CO_2_**
A_sat_	0.96	0.86	0.22	0.00	0.00	0.11
gs	0.23	0.99	0.48	0.08	0.00	0.03
R_10_	0.26	0.76	0.09	0.06	0.00	0.14
E_0_	0.17	**0.02**	0.08	0.07	0.19	0.11
Q_10_	0.21	**0.04**	0.10	0.06	0.16	0.11
Acclim_set-temp_		0.06		-	0.27	-
R/A	**0.04**	0.75	0.98	0.24	0.01	0.00
TSS	0.11	0.05	0.32	0.12	0.16	0.05
Starch	**0.01**	0.67	0.62	0.26	0.01	0.01
PEPc	0.79	**<0.001**	**<0.001**	0.00	0.19	0.35
COXII	**<0.001**	**<0.001**	**<0.001**	0.17	0.20	0.32
Mit. Number	**0.00**	0.32	0.48	0.32	0.03	0.02
Mit.Size	**<0.001**	**<0.001**	**0.06**	0.41	0.24	0.00

## Data Availability

All data are presented in the text.
